# Experiences of parents of an adolescent with intellectual disability in Giyani, Limpopo province, South Africa

**DOI:** 10.4102/hsag.v26i0.1538

**Published:** 2021-04-15

**Authors:** Tsakani Chauke, Marie Poggenpoel, Chris P.H. Myburgh, Nompumelelo Ntshingila

**Affiliations:** 1 Department of Nursing, Faculty of Health Sciences, University of Johannesburg, Johannesburg, South Africa; 2 Department of Educational Psychology, Faculty of Education, University of Johannesburg, Johannesburg, South Africa

**Keywords:** adolescent, experiences, intellectual disability, parents, South Africa

## Abstract

**Background:**

Parents of adolescents with intellectual disability experienced stress caused by challenges that come from having such adolescents. Those challenges affected the parents physically and emotionally, depending on the severity of the adolescent’s intellectual disability. Having an adolescent with an intellectual disability becomes a burden if the challenges were not resolved.

**Aim:**

This study aimed to explore and describe the experiences of parents of adolescents with intellectual disability in Giyani.

**Setting:**

This study was conducted at the participants’ homes in Giyani, Limpopo province, South Africa.

**Methods:**

A qualitative, exploratory, descriptive and contextual design was utilised. The main question was ‘How is it to have an adolescent with intellectual disability?’ Eight purposively sampled parents participated, and data were collected through in-depth interviews, observations and field notes. Data were analysed by means of thematic coding and an independent coder was consulted.

**Results:**

Four themes were identified. The study revealed that parents of adolescents with intellectual disability experienced negative emotional responses. Most parents reported a lack of support from family members and the community. They also reported caring challenges, yet some showed positive coping mechanisms.

**Conclusion:**

Parents of adolescents with intellectual disability experienced various challenges in caring for these children. A collaborative approach from relevant stakeholders could have a positive impact in supporting the parents of adolescents with intellectual disabilities.

## Introduction

Intellectual disability is a disorder that begins with the onset of a child’s developmental period, from childhood to adolescence (American Psychiatric Association [APA] 2013:664). It includes both intellectual and adaptive functioning deficits in conceptual, social and practical domains (APA 2013:663–666). Intellectual disability, also known as a general learning disability and mental delay, is a generalised neurodevelopmental disorder characterised by significantly impaired intellectual and adaptive functioning (National Institute for Health and Care Excellence [NICE] Guideline 2015:n.p.). It is defined by an Intelligence Quotient (IQ) score under 70, in addition to deficits in two or more adaptive behaviours including self-injury, stereotyped behaviour, aggression and destruction that affect every day, general living (NICE Guideline 2015:n.p.).

The *Children’s Act* No. 38 of 2005 defines a child as a person under the age of 18 years (Republic of South Africa 2006:20). In this study, the authors defined the adolescent as an individual in the 10–19-year age group (World Health Organization [WHO] 2014:n.p.). It has been estimated that 1% – 2% of the world’s population have intellectual disabilities (Maulik et al. 2011:32). Given the current global fertility rate of 2.5%, this suggests that globally, 1 in 50 parents have a child with intellectual disability (United Nations International Children’s Emergency Fund [UNICEF] 2011:1). A total of 90% of the world’s children aged 0–14 years live in low- or middle-income countries (World Bank 2012:3), and whilst intellectual disability has an estimated prevalence of between 2% and 3% in developed countries, a study in areas of South Africa found a prevalence rate of 3.6% (Foskett 2014:4). These estimates suggest that South Africa has a higher prevalence of intellectual disability compared with developed countries.

Kromberg et al. (2008) conducted a study in Bushbuckridge (which is in the northeast part of South Africa, Mpumalanga Province) to identify and describe childhood disabilities occurring in a rural South African population. Bushbuckridge has a similar setup as Giyani in Limpopo both being characterised by villages. In that study, children were screened in their homes in eight villages, and 3.6% of the children had an intellectual disability. No data were available on the prevalence rate of intellectual disability in Giyani, Limpopo province.

Children with intellectual disabilities are cared for by their parents who serve as their most constant and life-long caregivers (Families Special Interest Research Group [SIRG] Position Paper 2014:422); they play a critical role in shaping the development and life experiences of their children with intellectual disabilities. Intellectual disability in children during adolescence can be very disturbing, requiring proper attention, help and support from parents. Thus, parents of adolescents with intellectual disabilities have additional responsibilities and roles in caring for them. A parent is defined as the natural (biological) parent, whether they are married or not, and any person who, although not a natural parent, has parental responsibilities for a child or young person (Bedford Bough Council 2018:n.p.). It is also any person who, although not a natural parent, has to care for a child or young person (Bedford Bough Council 2018:n.p.). In this study, ‘parents’ refer to the biological mother or father or anybody who assumes that role, in caring for an adolescent with intellectual disability. In many contexts, parents’ involvement extends over their life span, with siblings and extended family members taking on caring roles, especially when parents are no longer able to do so (UNICEF 2011:464; WHO Regional Office for Europe 2010:14). The International Human Rights Conventions require member states of the United Nations to make an effort in ensuring that when a parent cannot care for a child, alternative arrangements are made, either with the child’s extended family or in substitute family settings in the community (UNICEF 2011:465). The continuing reliance in some countries on institutional care for many children with intellectual disabilities is a source of major concern (UNICEF 2011:465; WHO Regional Office for Europe 2010:14).

In certain cultures, parents of a child with disabilities may differ from parents who do not have a child with disabilities, in various important ways (UNICEF 2011:465). These include an increased risk of exposure to socio-economic disadvantage, exposure to disability-related discrimination, coping with exceptional and prolonged caring tasks, complex interactions with disability services and continuing to provide support into later childhood, adolescence, and adulthood. Parents’ experiences are likely to vary as a function of their social and cultural context, and the nature and severity of the child’s impairment (UNICEF 2011:465). In addition, because of the lack of reliable statistics, there is a paucity of published literature on intellectual disability in South Africa. The lack of evidence-based publications precludes an accurate description of the prevailing epidemiology and burden of intellectual disablement in South Africa (Foskett 2014:5).

Caring for an adolescent with intellectual disability can be challenging, demanding and filled with pressure (Lafferty et al. 2016:29). Parents are seldom able to meet the additional financial burden of regular visits to hospitals, additional expenses for equipment and assistive devices and other necessities (Foskett 2014:9). Parents’ caregiving in the field of intellectual disability has received increased attention over the last few years, yet relatively little is known about caregiving demands, family relationships, family support and compound caregiving prioritisation. If parents are to be supported in their caring role, it is important for health professionals to develop a better understanding both of the demands they face and of the mechanisms that allow them to continue their role in caring for their adolescent with intellectual disabilities (Lafferty et al. 2016:26).

Whilst there is a paucity of published literature on intellectual disability in South Africa, there is extensive literature internationally that examines the stress on parents of adolescents with intellectual disabilities who care for them. One study conducted by Lee (2013:4255) in the United States confirmed that parents of children with intellectual disabilities report clinically significant levels of stress, anxiety and depression. Similarly, in another study conducted in Canada by McConnell and Savage (2015:101), it was stated that chronic stress may also be the underlying cause of the increased risk of families of children with intellectual disabilities who experience marital disruption and family dysfunction. These studies provide an indication of the mental health challenges of parents of children with intellectual disability. There is a gap in the description of the experiences that these parents endure in South Africa, and it is imperative that the experiences of these parents be recognised to plan for the needs of those with intellectual disability across their lifespan (Adams 2010:436). The research question that arose from this problem statement was: What are the experiences of parents of adolescents with intellectual disability in Giyani?

## Aim

The aim of this study was to explore and describe the experiences of parents of adolescents with intellectual disability in Giyani.

## Research methods and design

### Design

A qualitative, exploratory, descriptive and contextual design was applied in this study (Creswell 2014:4) to capture the essence of the experiences of parents of adolescents with intellectual disability. Information was collected as it was expressed naturally by participating parents. The researcher asked open-ended questions so that participants could describe their experiences as they viewed and lived them. Qualitative research was conducted to gain an understanding and discover meaning about the experiences of parents of an adolescent with intellectual disability.

### Research setting

The study was conducted in Giyani, Limpopo province, South Africa. Greater Giyani Municipality is one of five local municipalities falling within Mopani District Municipality in Limpopo Province. Giyani has 10 traditional authority areas comprising 91 villages (Greater Giyani 2020:n.p.). The Greater Giyani Municipality has a population of 244 217 people (Statistics South Africa [STATSSA] 2012).

### Population and sample

Rebar and Gersch (2015:110) described the population as the entire group of individuals about whom the researcher is interested in gaining knowledge. The population in this context comprised parents who had an adolescent with intellectual disability. It was not possible to determine the size of the population and include all parents of an adolescent with an intellectual disability in the study, thus a sample had to be drawn. A sample is a subset of the total group of interest in a study (Rebar & Gersch 2015:31). A purposive sampling method was used, which is also known as purposeful, judgemental or selective sampling because it entails that the researcher makes a judgement about the population to be studied (Gray, Grove & Sutherland 2017:345).

Participants had to meet the following inclusion criteria:

- Adolescent with intellectual disability admitted to a unit in a psychiatric hospital.
- Parents could either be a male or female parent of an adolescent with intellectual disability.
- The adolescent had to be between the ages of 15 and 17 years. These ages were chosen because they are a critical period during which individual differences may be heightened.
- Participants preferably had to be able to communicate in English, Xitsonga, Sepedi or Tshivenda; these are the commonly used languages in Giyani.

### Ethical considerations

The researchers obtained ethical approval (Ref: REC-01-90-2019) from the Research Ethics Committee, Faculty of Health Sciences at a University in Gauteng, Johannesburg, before commencing the study. Ethical approval was also obtained from the Limpopo Provincial Government dated 31 October 2018 and the Homu Traditional Council dated 26 September 2018. In addition, the ethical principles of autonomy, beneficence, non-maleficence and justice (Dhai & McQuoid-Mason 2011:14–15) were applied throughout the study.

Autonomy means that the study participants must be allowed to make a free, independent choice to participate in the study. Participants had the right to self-determination, meaning that the participants were autonomous and had the right to make a knowledgeable decision free from coercion whether to participate in research or withdraw from the study (Rebar & Gersch 2015:136). In this study, autonomy was ensured by providing the participants an information letter, which was written in their preferred language. This information letter indicated the purpose and benefits of participating in the research. The participants were made aware of their right to refuse to participate in the study. The participants gave permission for the interviews to be audio-recorded. The participants were informed that they would be provided with a number or code to ensure that their identities are not recognised and will be kept anonymous.

According to Dhai and McQuoid-Mason (2011:14–15), researchers have a responsibility to ensure that participants are not unduly influenced to participate in research. The researcher ensured that the participants fully understood what informed consent entails for them to make an informed decision. The participants determined a convenient time for the interviews to be conducted.

The principles of beneficence and non-maleficence state that the benefits of participating in a study should outweigh the risk for the individual and wider society (Holloway & Wheeler 2010:303). Therefore, the researcher ensured that during this study, the participants were protected against any kind of harm and potential risk to their psychological well-being, mental health, personal values and dignity. The participants were not harmed during the interviews, as all necessary precautions were taken not to harm them. There were no direct benefits to the participants. All participants were respected and treated equally and had an opportunity to ask questions relating to the study. The participants’ emotional safety was ensured by the researcher reassuring them that their views would be respected and that participation was voluntary to ensure informed consent.

### Data collection

Data collection was carried out through individual in-depth interviews, observations and field notes (Holloway & Galvin 2017:3–11). The researcher (T.C.) conducted the in-depth interviews. T.C. is a registered psychiatric nurse who has completed her clinical component of a Masters in psychiatric mental health nursing. The researcher has the skills and knowledge to conduct individual, in-depth interviews and had rapport with participants. The interviews were conducted from November 2018 to January 2019 at the homes of the participants. The researcher posed one main, open-ended question to the participants:

### How is it to have an adolescent with intellectual disability?

All the in-depth interviews were conducted in Xitsonga because participants preferred to be interviewed in their home language. Follow-up questions, probing and minimal verbal responses were used only to confirm or look for the underlying meaning of statements given by the participants (De Vos et al. 2011:345). Permission was requested from the participants to audio-record the interviews. The interviews lasted for 40–60 min. Data collection was stopped when enough rich, meaningful data had been obtained to achieve the objectives of the study, which is called data saturation. Data saturation was reached at the sixth interview, but two additional interviews were conducted to confirm the themes. Gray et al. (2017:255) defined ‘data saturation’ as the point at which new data become redundant with what has already been found and no new themes can be identified. The researcher observed and wrote observations and field notes after each in-depth interview.

### Data analysis

Data analysis includes both the coding and the thought processes required in assigning meaning to data. The researcher sought meaning from all the raw data and used Tesch’s method of thematic data analysis (Creswell & Poth 2018:196) to analyse and make sense of data that were collected. The researcher transcribed all the in-depth interviews then read and re-read these whilst making notes. Units of meaning were identified from the data and transcribed interviews and field notes were linked together to form themes. The three co-authors examined the transcribed in-depth interviews before handing over to an independent coder. An independent coder who is experienced in qualitative research then analysed the data. The researcher and the independent coder met for a consensus discussion on the results of the data analysis. The researcher and the independent coder agreed on the final themes.

### Measures of trustworthiness

The researcher ensured the trustworthiness of the study by applying the measures of credibility, transferability, dependability and confirmability (Lincoln, Lynham & Guba 2011:100).

Credibility focuses on whether the results accurately represent the underlying meaning of data, and it is improved by prolonged engagement in the data collection process and triangulation (Houser 2012:425). Credibility was maintained through prolonged engagement; the researcher spent 2 months in the field. Data were also independently analysed by the researcher and an independent coder. In this study, the researcher used in-depth interviews, observations and field notes to ensure data triangulation.

Lincoln et al. (2011:102) described ‘transferability’ as the extent to which the findings of a study are confirmed by or applicable to a different group in a different setting (Rebar & Gersch 2015:155). A dense description of the research methodology and the participants’ demographics has been provided to ensure the transferability of this study, along with supporting direct verbatim quotes from the participants.

Lincoln et al. (2011:102) used the term ‘dependability’ instead of reliability, which means that the research findings should be consistent and accurate to establish the trustworthiness of the study (Holloway & Wheeler 2010:55). The researcher collected data until data saturation was achieved. This was the point where there were repeated and consistent findings from the in-depth interviews.

Moreover, according to Lincoln et al. (2011:102), ‘confirmability’ means that an audit or decision trail is necessary for readers to trace data to their sources. This was accomplished by incorporating an audit procedure, and an audit of the entire research process was carried out to ensure confirmability. The researcher kept documentation of decisions about the data collection and analysis process. Documentation from the audit trail included field notes about the collected data, ideas developed during the analysis or notes regarding approaches to categorise or organise the data. Confirmability was ensured through ongoing analysis through member checking after confirming the correctness of their responses with participants.

## Findings

Eight parents of adolescents with intellectual disability in Giyani were interviewed. Two were the adolescents’ grandmothers, four were biological mothers of the adolescent with intellectual disability, one was an aunt and one was a sister. The demographics of the participants are summarised in Table 1.

**TABLE 1:** Demographics of participants.

Participant number	Age (years)	Gender	Relationship	Socio-economic status	Housing	Years looking after adolescent
1	57	Female	Grandmother	Domestic worker	RDP house	15
2	51	Female	Aunt	Unemployed	Four-roomed house	4
3	64	Female	Mother	Pensioner	RDP house	17
4	55	Female	Mother	Domestic worker	Cracked RDP house	17
5	34	Female	Sister	Casual employee	RDP house	10
6	48	Female	Mother	Casual employee	Four-roomed house	16
7	46	Female	Mother	Employed	Five-roomed house	17
8	76	Female	Grandmother	Pensioner	RDP house	17

*Source*: Adapted from Chauke, T., 2019, ‘The experiencs of parents who have an adolescent with intellectual disability in Giyani’, Masters thesis, University of Johannesburg, Johannesburg, viewed 27 March 2020, from http://hdl.handle.net/10210/412128.

RDP, RDP stands for Reconstruction and Development Programme which is a South African socio-economic policy implemented by the government of President Nelson Mandela in 1994.

Parents of adolescents with intellectual disability faced several challenges. The main challenge was stress, and the four themes that were developed emanated from the stress the parents were experiencing.

- The first theme: This theme is about emotional responses experienced by parents of adolescents with intellectual disability. Parents had emotional responses that were manifested by being sad, distressed, worrying, being fearful of death and the future and feeling that they were neglecting other family members.
- The second theme: This theme is about lack of support experienced by parents of adolescents with intellectual disability. Most participants complained of being neglected by family members and abandoned by their husbands because of the adolescent with intellectual disability, social isolation and stigma.
- The third theme: It is about caring challenges experienced by parents of adolescents with intellectual disability. Participants experienced caring challenges as some of the adolescents were aggressive and destructive and some were difficult to manage because of their physical body deformity and behaviour, which was distressing to the parents. These caring challenges ranged from physical frailty, difficulties in managing the adolescent and poverty, as they had financial constraints and inadequate living environments.
- The fourth theme: It is about positive coping mechanisms experienced by parents of adolescents with intellectual disability. Generally, they coped positively by putting their faith in God and accepting their situation. The discussion of the findings was guided by the themes presented in Table 2.

**TABLE 2:** Themes of the experiences of parents of adolescents with intellectual disability.

Themes
Emotional responses experienced by parents of adolescents with intellectual disability.
Lack of support experienced by parents of adolescents with intellectual disability.
Caring challenges experienced by parents of adolescents with intellectual disability.
Positive coping mechanisms experienced by parents of adolescents with intellectual disability.

*Source*: Adapted from Chauke, T., 2019, ‘The experiencs of parents who have an adolescent with intellectual disability in Giyani’, Masters thesis, University of Johannesburg, Johannesburg, viewed 27 March 2020, from http://hdl.handle.net/10210/412128.

### Theme 1: Emotional responses experienced by parents of adolescents with intellectual disability

When parents were interviewed, it was discovered that they had developed emotional responses, which included sadness, worry, fear of death and the future, distress and feelings that they were neglecting other family members.

Most parents experienced sadness caused by the challenges they encountered because of having an adolescent with intellectual disability. Some of these adolescents’ biological parents had died, thus their siblings or grandmothers had to take over and were left to tell the story. Some explained that they became tearful when they visited the adolescent at the hospital and were distressed after the visit. They said:

‘But most of the time I cry when I see her, and when she sees me cry, she will also become sad.’ (Participant 1, female, 57 years old, grandmother)

‘When something doesn’t go well, I sit down and cry alone; who shall I tell? When something doesn’t go well, I sit down and cry. While crying that I have no parents, and on one side my sibling has a disability, on the other side, nothing is working out for me.’ (Participant 5, female, 34 years old, sister)

Continuous sadness could potentially change the mental health status of parents who have an adolescent with intellectual disability. One of the parents explained how her mental health was altered:

‘So, my problems gave me a short temper. I’m no longer the same old person. Emm, it is like that, my patience is so short.’ (Participant 7, female, 46 years old, mother)

Parents were worried about what would happen to their adolescent if they died before the adolescent. They were also concerned about the adolescent’s future. They were anxious about who would take over in caring for the adolescent when they died because they lacked support whilst they were still alive. One of the parents explained:

‘What I pray for day and night is that before I die, God keep me away from death until I bury her. She should pass on before me so that I will be able to bury her; then God may let me die.’ (Participant 1, female, 57 years old, grandmother)

One parent who was dependent on the adolescent’s social grant indicated that she understood that the grant was helping them, but she was bound to take the adolescent to the hospital, knowing that the grant would be cut off. She was worried about the safety of the adolescent when she was not around, as she had to work. She also showed concern about sexual harm and exploitation of the adolescent and the possible consequences of a pregnancy. She shared:

‘My concern is for the safety of my younger sister. This world is cruel now. You will be surprised to find her raped.’ (Participant 5, female, 34 years old, sister)

Another parent feared that her child would no longer be visited if she passed on because no one was visiting the adolescent except her. She said:

‘And when she is in the hospital, there is no one who visits her except me, meaning that if I die, she will have no one, there will be no one to visit and look after her.’ (Participant 4, female, 55 years old, mother)

Parents explained how distressing it was to have an adolescent with intellectual disability as the adolescent was partially or fully dependent on them to meet the activities of their daily living. One parent said those who were taking care of the adolescent passed on. As a result, she had to take over the caregiving role of her sister. She wished someone else was alive to ease her burden. The following direct quotation presents her view:

‘Why are they all gone? It would have been better if it was one parent who passed away and one left … my mother is gone and my father too, but why did my grandmother also pass away? My uncle’s wife took care of her … she also passed away.’ (Participant 5, female, 34 years old, sister)

This participant continued indicating that she had challenges living with the adolescent (her sibling), as her other siblings were also staying with her because their mother passed on. In addition, she was married and had her own family:

‘I stay with them all. Eh … thus it is a challenge to take the adolescent to stay with me, I’m with my other two siblings. They also need me, and my two children too.’ (Participant 5, female, 34 years old, sister)

### Theme 2: Lack of support experienced by parents of adolescents with intellectual disability

Parentsand their adolescents with intellectual disability, faced challenges of being neglected and rejected by family members and they experienced social isolation and stigma (Chauke et al. 2019:46).

Family members tended to ignore the parents who had an adolescent with intellectual disability and the adolescents themselves. Some stated that if they had support from family members, the situation might have been different. This was what one of the parents said:

‘I have never seen my siblings come with a 12.5kg bag of mealie meal. I have never received support from my siblings, nor have they visited me. They work and they are successful.’ (Participant 1, female, 57 years old, grandmother)

It seemed as if the husbands of the mothers of adolescents with intellectual disability, or the fathers of those adolescents, could not stand to have such a child. Many of the mothers were rejected by the fathers of these adolescents because of the latter’s intellectual disabilities. Participants shared:

‘His father is alive. His father kicked them away when he was a toddler, saying to his mother that she must leave the house because she had given birth to a child with disability. He no longer knows his father. His father kicked them out of the house when the child learned to sit, and his father realised that the child seemed to have a disability.’ (Participant 2, female, 51 years old, aunt)

‘Her father has never minded about her since he realised she has an intellectual disability.’ (Participant 3, female, 64 years old, mother)

Some community members seemed to disapprove of intellectual disability, mostly the severe and profound forms of disability. The family and the community members distanced themselves from the parents of an adolescent with intellectual disability. One participant explained that family members seemed to be uncomfortable living with the adolescent with intellectual disability, perhaps because they did not know how to treat her within the family. She said:

‘It was Good Friday when I brought her here to visit me. I realised that it made people uncomfortable. When they … they don’t understand how they should live with her. It’s only me who is able to live with her because she is my sibling. Yes, they don’t understand how they should treat her, how they should live with her. They don’t understand.’ (Participant 5, female, 34 years old, sister).

### Theme 3: Caring challenges experienced by parents of adolescents with intellectual disability

Caring challenges included physical frailty of the parent, difficulties in managing the adolescent, lack of physical support and poverty (Author 2020:57). A majority of the parents complained about physical health problems. Some parents indicated that they were no longer able to care for their adolescents because of their physical challenges. Participants explained:

‘Surely, I cannot do it, I can’t do it. I am not able to take care of her. I can’t carry her anymore; I can fall with her. My legs make it difficult for me; they are painful.’ (Participant 1, female, 57 years old, grandmother)

‘When you change her napkin you have to struggle, she refuses, she … she is difficult altogether, I have to struggle with her. And now I’m becoming old and sick … yes, I don’t know. When I say I’m sick, I mean sometimes I feel dizzy, is it not because of my heart?’ (Participant 4, female, 55 years old, mother).

The adolescent could be unmanageable depending on the severity of their intellectual disability – some needed carers with physical strength. A few parents explained how difficult it was to care for an adolescent with intellectual disability at home, as they had to bathe them, feed them, change diapers and supervise them. They said:

‘He was difficult. When we bathe him, it was not easy; he would beat us and bite himself. You find that when you give him food, he will hit his head against the floor. When his mother was still alive he would beat her.’ (Participant 2, female, 51 years old, aunt)

‘The problem is that she cannot do anything for herself, she can only feed herself. She needs to be bathed, for her soiled napkin to be changed, and I can’t pick her up into the bath alone. All she can do is sit. She can’t speak.’ (Participant 6, female, 48 years old, mother).

The caring challenges experienced by the parents of adolescents with intellectual disability were coupled with a lack of resources. Participants faced the challenge of a lack of physical resources to support their adolescent with intellectual disability. Some of the adolescents needed accessories such as wheelchairs that parents could not afford, so they ended up carrying the adolescent on their backs or in their arms, which could be heavy for them and strained their body. Some parents said:

‘I cannot take care of her. I can’t carry her anymore. I can fall down with her ….’ (Participant 1, female, 51 years old, grandmother)

‘When I take him to the hospital, I carry him on my back, or carry him in my arms with the help of somebody.’ (Participant 2, female, 51 years old, aunt)

Most parents had financial difficulties and inadequate housing. Financial strain added to the stress that these parents already had because their adolescents’ special needs could increase their financial burden. Some participants explained how they make a living:

‘So, there is poverty within the family. I was married but my marriage was useless ….’ (Participant 3, female, 64 years old, mother)

‘That’s why I applied at that hospital for her, and they explained that they would cut off her grant. Though it was helpful for my siblings to be stable and for us to stay together, because I was using the grant to buy them food and other things, but I told them I have no choice.’ (Participant 5, female, 34 years old, sister)

Housing was another problem for parents who had an adolescent with intellectual disabilities. One parent said:

‘That side where there is that cement slab, there was a cracked two-roomed house where we used to sleep. During thunderstorms, my children used to go to our family friend for shelter.’ (Participant 1, female, 57 years old, grandmother)

Another shared:

‘I stay in a three-roomed house which has cracks all over. It is cracked because it was built with mud bricks.’ (Participant 4, female, 55 years old, mother)

### Theme 4: Positive coping mechanisms experienced by parents of adolescents with intellectual disability

Parents were coping with their situation by putting their trust in God and gradually accepting their adolescents’ condition (Author 2020:65). In all the interviews, parents indicated that it was only God who kept them going. Participants indicated their trust in God as follows:

‘So, I will never kill my child; she is a gift from God. I don’t know His intentions in giving me this child. I have to accept it. She is my child.’ (Participant 4, female, 55 years old, mother)

‘This is what God has given me. I believe that this is how God wants my life to be.’ (Participant 5, female, 34 years old, sister)

Parents, who had an adolescent with intellectual disability, tended to accept the adolescent’s condition as they had to live with them for the rest of their lives. Although it was not easy, with time they became used to this situation. Some of the participants showed acceptance by explaining:

‘I have explained that this does not mean life has to end here. Just because our parents died and left me with my siblings, one who has an intellectual disability, life has to go on.’ (Participant 5, 34 years old, sister)

‘I am not ashamed to walk with her. I will not lie about that. I can travel with her using public transport. I have accepted that I have a sibling with intellectual disability and now I’m her mother.’ (Participant 5, female, 34 years old, sister)

## Discussion

This study provided some insight into the experiences of parents of adolescents with intellectual disability in Giyani. Four key themes were identified, emotional responses, lack of support, caring challenges and positive coping mechanisms.

The themes that were identified in this study suggest the core challenges that the parents of adolescents with intellectual disability experience. The themes also indicate the coping mechanisms used by the parents. Literature highlighted that expectations often turn into some level of disappointment if a child has an impairment, as communities perceive a child with impairments as being cursed by God (Tederera & Hall 2017:78). This explains why parents became saddened by the fact that they had adolescents with intellectual disability, which they had not expected. According to Willingham-Storr (2014:7), some researchers identified that parents who have an adolescent with intellectual disability were worried about future provision for their children and talked about the prospect of their death as a hidden concern. It was confirmed in this study that parents worried about what would happen to the adolescent when they were no longer around. Badu (2016:20) agreed that it is distressing to take care of a sibling with intellectual disabilities whilst having a family of one’s own. Lafferty et al. (2016:22) supported this theme by saying that in some instances, a sibling becomes the primary caregiver for their brother or sister with intellectual disabilities when a parent dies. They further claim that siblings may find themselves trying to balance the constant demand of caring for their brother or sister with intellectual disabilities, whilst simultaneously trying to raise a young family of their own.

Some communities perceive intellectual disability as a punishment for what one has performed wrong. This would perhaps explain the minimal support received from the community (Tederera & Hall 2017:283). Extended family members may be unwilling to contribute to and support parents in raising an adolescent with intellectual disability for fear of associated discrimination and stigmatisation (Resche et al. 2010:139). This view was confirmed in this study when participants reported family members and the community distanced themselves from parents of adolescents with intellectual disability. In this study, the lack of support was possibly attributed to a lack of understanding and knowledge of intellectual disability.

McNally and Mannan (2013:2) supported the view that rejection by fathers is common; spousal and immediate family support is, therefore, absent, and the onus of care falls on the mother. Mudhovozi, Maphula and Mashamba (2012:148) also emphasised that in Limpopo, South Africa, the burden of raising a child with intellectual disability lies with the mother. Similarly, Harper et al. (2013:14) indicated that women are often abandoned by their husbands and have to bear the brunt of caring for the child with intellectual disability alone. This study’s findings also confirm existing claims of women being rejected and abandoned by their husbands and the fathers of their children. The lack of support by family and community members seemed to predispose participants to stigma and discrimination (Tederera & Hall 2017:3). Gupta, Mehrotra and Mehrotra (2012:41) indicated that the stigma associated with intellectual disability is so pervasive that support from the family and society is limited.

The participants articulated the caring challenges they experienced with their adolescents with intellectual disability. The parents experienced physical frailty amongst the challenges. The somatic and psychological health, emotional health, quality of life and well-being have all been demonstrated to deteriorate amongst parents of adolescents with intellectual disabilities (Wittenberg & Prosse 2013:489). Because of increased parental responsibilities, parents of adolescents with intellectual disabilities can be at high risk of experiencing depression, physical health problems and decreased quality of life (Resche et al. 2010:139). In this study, the caring challenges were coupled with the ageing process of parents of adolescents with intellectual disability.

Parents had to deal with numerous physical, behavioural and developmental issues associated with adolescents with intellectual disabilities, such as providing personal care, for example, toileting, eating, bathing and helping females during menstruation (Badu 2016:24). Similarly, in this study, the participants acknowledged the difficulties of caring for an adolescent with intellectual disability such as bathing and feeding. These parents suffered from exhaustion and stress because of the intensity of care required. Feeding, clothing, bathing and diapering an infant are much easier than doing the same task for someone who weighs more than 80 pounds (36.28 kg) (Thwala, Ntinda & Hlanze 2015:207). Previous studies in this area have shown that services do not always address the needs of parents and do not offer continual support (Willingham-Storr 2014:4). Challenges in parenting adolescents with intellectual disabilities are also encountered because of a lack of resources related to daily living support (Resche et al. 2010:139). Thwala et al. (2015:208) agreed that raising a child with intellectual disabilities might be more expensive than raising a typical child. Exacerbating these financial challenges is the finding that children with intellectual disabilities are significantly more likely to live in families considered to be poor, and an adolescent with intellectual disability might also increase the risk of the family being poor (Resche et al. 2010:139). Literature indicates that economic difficulties experienced by the parents affected the provision of care for the adolescent, including challenges in sustaining basic needs, education and housing (Mbwilo, Smide & Aarts 2010:10). The findings in this study that caring challenges are combined with a lack of resources are thus substantiated.

The participants articulated positive coping mechanisms. Ha, Grenberg and Seltzer (2011:406) stated that amongst people of African descent, including parents of children with intellectual disabilities, spirituality and family support systems are frequently relied on as coping strategies in times of adversity. Parents emphasised their belief in God and His power to carry them through their experiences of caring for an adolescent with intellectual disability (McNally & Mannan 2013:4). According to Heather and Aldersey (2012:37), many parents take comfort in their spirituality, which helps them view their child as a blessing or a test of their faith, rather than a burden. Despite the increased level of stress experienced by parents of adolescents with intellectual disability, many parents of such adolescents are well-adapted and appear resilient in the face of challenges (Heather & Aldersey 2012:3). The findings of this study provided important insights into how parents of adolescents with intellectual disability accepted and put God first to cope with their difficulties.

### Study limitations

In this study, the participants were limited in the sense that only those who had an adolescent between the age of 15 and 17 years were interviewed. This was carried out to focus on a specific group of parents. All the participants were female because the male parents were not available as most had rejected the adolescent and their mothers. Their views were thus not included in this study, and the study therefore does not reflect the views of all parents of an adolescent with an intellectual disability.

### Implications and recommendations

The findings of this study indicated that something needs to be carried out for parents of adolescents with intellectual disabilities. The family and community members need to support those parents. With support from family, the community, health and social services, parents might be able to better cope with their situation and their burden of caring for an adolescent with intellectual disability might be reduced. If parents are to be supported in their caring role, health professionals should develop a better understanding of both the demands they face and of the mechanisms that allow them to continue their role in caring for their adolescents with intellectual disabilities. Health and social services should also intervene and address these parents’ challenges. The community needs to be aware of mental health conditions such as intellectual disability.

Some parents indicated that they were very pleased to be visited by the researcher because no one from the institution had visited them to hear how they feel. Home visits and support groups for parents of adolescents with intellectual disabilities should be facilitated by psychiatric nurses. Parents, family members and the community should receive health education to be empowered about intellectual disability to provide the necessary support to affected individuals. These recommendations to support the parents of adolescents with intellectual disability are important because challenges experienced by these parents can reduce their capacity to provide the necessary care and treatment for their adolescents (Bourke-Taylor, Howie & Law 2010:128).

## Conclusion

Parents of adolescents with intellectual disability experience various challenges in caring for their children. It is therefore fundamental for all stakeholders with an interest in parents of adolescents with intellectual disabilities to collaborate and have a better understanding of their challenges. Healthcare professionals, especially professional nurses, should take an active role in reducing the burden of care for parents of adolescents with intellectual disabilities.
